# Magic Healer, Not a Physician!!

**Published:** 1908-05

**Authors:** 


### MAGIC HEALER, NOT A PHYSICIAN! !

Of far-reaching significance to the medical profession is the recent decision of the court of appeals, that a "Magic Healer" is not a physician in the sense of being required to pay license to practice medicine in the State.

Judge B. H. Hill rendered this decision in the case of Bennett v. Ware, which was appealed from the city court of Fitzgerald.

A. D. Bennett advertised himself as a "magic healer," stating that he could cure by laying his hands on the diseased or sick persons, and that he had "magic power given direct from the Lord."

D. B. Ware swore out a warrant charging him with practicing as a physician without having paid his license fee. Upon preliminary hearing Bennett was discharged from custody, and thereupon brought suit for damages against Ware for malicious prosecution. Bennett lost his suit in the lower court, and the court of appeals upholds that decision.

To hold that such an individual as Bennett is not to be regulated by the medical practice statutes of Georgia, to all intents and purposes, renders the present medical laws of the state worthless and opens wide the gate for quacks and charlatans of all descriptions to swoop down upon us, unhampered by any restrictions.

At the present time when so many important measures aside from drugs, are being used by regular physicians in curing disease and when *diagnosis* is recognized as absolutely necessary to successful practice, not only by physicians but by laymen of ordinary common sense, it seems incomprehensible that Judge Hill should have handed down such a decision. Is not a physician practicing medicine who recognizing incipient tuberculosis, advises the open air treatment and liberal feeding, without the administration of any medicine whatsoever? Is not the dermatologist practicing medicine who cures a cancer with X-ray? Is there a measure used by physicians or quacks with which greater harm can be done to a patient than with the X-ray ignorantly used? Shall we allow charlatans who know nothing of disease and less of such a curative measure to slaughter trusting patients with it? Shall osteopathists and Christian Scientists be allowed to treat patients for ailments they cannot diagnose, simply because they do not administer drugs and medicine?

If the law is intended to prevent an incompetent physician from having charge of the sick is it not all the more important that it should prevent an admitted imposter from assuming these duties? It is a strange law that intends to protect the people from incompetent medical attendance and yet does not apply to a quack who assumes the duties of a physician.

The law (according to Hill), renders *him* immune because he is *not* a physician. The very fact that he is not a physician makes the necessity of a law all the more urgent. According to Judge Hill's decision a lawyer may treat the sick, as long as he eschews medicine, not because he knows anything about the disease he has charge of, but because *he is a lawyer* and cannot be regulated by the law intended for physicians.

One would think there could be no debating the fact that the medical practice law should protect the sick from imposters no matter what their profession or methods. It is just as rational to expect a broken automobile to run by saying a few incantations over it as to think that an imposter can cure a patient by "laying on of hands." In each case the determination of what the trouble is, must be the first step in the correction thereof. Diagnosis is therefore an integral and essential part of the curing of disease regardless of the measures employed.

Such a decision will bring to our State a swarm of new "systems of practice," which no matter how absurd their claims or how dangerous their methods, cannot be affected by the law so long as they *use no drugs*.

Can you imagine a more illogical, intolerable position? Infectious diseases will be unrecognized, and therefore unchecked in their course of disaster and death. Patients will be robbed of their health as well as money and a premium placed upon cupidity and quackery. Is it not time for the medical profession to make an organized effort to obtain a sensible decision defining the practice of medicine?

